# The DNA/RNA-Dependent RNA Polymerase QDE-1 Generates Aberrant RNA and dsRNA for RNAi in a Process Requiring Replication Protein A and a DNA Helicase

**DOI:** 10.1371/journal.pbio.1000496

**Published:** 2010-10-05

**Authors:** Heng-Chi Lee, Antti P. Aalto, Qiuying Yang, Shwu-Shin Chang, Guocun Huang, Daniel Fisher, Joonseok Cha, Minna M. Poranen, Dennis H. Bamford, Yi Liu

**Affiliations:** 1 Department of Physiology, University of Texas Southwestern Medical Center, Dallas, Texas, United States of America; 2 Institute of Biotechnology and Department of Biosciences, University of Helsinki, Helsinki, Finland; 3 University of Texas, Austin, Texas, United States of America; Vanderbilt University, United States of America

## Abstract

The production of aberrant RNA (aRNA) is the initial step in several RNAi pathways. How aRNA is produced and specifically recognized by RNA-dependent RNA polymerases (RdRPs) to generate double-stranded RNA (dsRNA) is not clear. We previously showed that in the filamentous fungus *Neurospora*, the RdRP QDE-1 is required for rDNA-specific aRNA production, suggesting that QDE-1 may be important in aRNA synthesis. Here we show that a recombinant QDE-1 is both an RdRP and a DNA-dependent RNA polymerase (DdRP). Its DdRP activity is much more robust than the RdRP activity and occurs on ssDNA but not dsDNA templates. We further show that Replication Protein A (RPA), a single-stranded DNA-binding complex that interacts with QDE-1, is essential for aRNA production and gene silencing. In vitro reconstitution assays demonstrate that QDE-1 can produce dsRNA from ssDNA, a process that is strongly promoted by RPA. Furthermore, the interaction between QDE-1 and RPA requires the RecQ DNA helicase QDE-3, a homolog of the human Werner/Bloom Syndrome proteins. Together, these results suggest a novel small RNA biogenesis pathway in *Neurospora* and a new mechanism for the production of aRNA and dsRNA in RNAi pathways.

## Abstract 2

The *Neurospora* RNA-dependent RNA polymerase QDE-1 is an RNA polymerase that can use both RNA and DNA as templates, suggesting a new mechanism for small RNA production.

## Author Summary

Small RNA molecules (20–30 nucleotides) play important roles in many cellular processes in eukaryotic organisms by silencing gene expression. To generate the many forms of small RNAs, DNA is first transcribed to produce single-stranded RNA (ssRNA), which then is converted to double-stranded RNA (dsRNA) by an RNA-dependent RNA polymerase (RdRP). However, it is not clear how the ssRNA templates are synthesized from DNA and specifically recognized by RdRPs amidst a sea of single-stranded, cellular RNAs. We previously showed that in the filamentous fungus *Neurospora* the production of one type of small RNA called qiRNA, which is specifically induced after DNA damage, requires the RdRP QDE-1. Here, we investigated the precise contributions of QDE-1 to the synthesis of ssRNA and dsRNA. We show that QDE-1 is surprisingly promiscuous in its template choice in that it is able to synthesize RNA from both ssRNA and single-stranded DNA (ssDNA). These results suggest that QDE-1 first generates ssRNA from a DNA template and then converts the ssRNA into dsRNA; this combination of activities in one protein ensures the specific action by RdRP on aberrant RNA in lieu of other single-stranded cellular RNA. In addition, we identified Replication Protein A, a ssDNA-binding protein that interacts with QDE-1, as an essential factor for small RNA production. Furthermore, we were able to reconstitute synthesis of dsRNA from ssDNA in a test tube using purified QDE-1 and RPA proteins, demonstrating the ability of this relatively simple biosynthetic system to generate the nucleic acid trigger for gene regulation. Together, these results uncover the details of a new and important small RNA production mechanism in cells.

## Introduction

RNA interference (RNAi) refers to a group of post-transcriptional or transcriptional gene silencing mechanisms conserved from fungi to mammals [1]–[6]. The RNAi pathway is triggered by the presence of double-stranded RNA (dsRNA), which is cleaved by the ribonuclease-III domain-containing enzyme Dicer to generate 20–25 nucleotide long small interfering RNA (siRNA) duplexes. siRNA is then loaded onto the RNA-induced silencing complex (RISC), in which an Argonaute (Ago)-family protein, guided by the siRNA, mediates the cleavage of homologous RNAs.

In fungi, plants, and *Caenorhabditis elegans*, the production of endogenous dsRNA and siRNA requires RNA-dependent RNA polymerases (RdRPs), which can generate dsRNA using single-stranded RNAs (ssRNAs) as templates [1],[2],[7]–[10]. Some RdRPs are also important for the amplification of dsRNA and siRNA [11],[12]. However, the production mechanism of the initial ssRNA templates used by RdRPs, often called aberrant RNAs (aRNA) or pre-siRNAs, is not well understood. In addition, the nature of aRNA and how RdRPs recognize aRNA specifically over other cellular RNAs are unclear.

In plants, the DNA-dependent RNA polymerase IV (Pol IV) is important for siRNA production and RNAi-directed transcriptional silencing [13],[14]. However, Pol IV homologs are not found in fungal or animal genomes. In fission yeast, mutants of the RNA polymerase II (Pol II) subunits show loss of centromeric siRNA and RNAi-dependent heterochromatin formation at the centromeric repeat regions, probably by coupling transcription with transcriptional silencing machinery [1],[15],[16]. It is not known whether Pol II plays a similar role in posttranscriptional silencing and in the silencing of other chromosomal regions.

In the filamentous fungus *Neurospora crassa*, quelling is a post-transcriptional silencing mechanism triggered by multiple copies of transgenes during vegetative growth [2]. In the quelling pathway, QDE-1 (QUELLING DEFICIENT-1, an RdRP), QDE-2 (an Argonaute protein), QDE-3 (a RecQ DNA helicase homologous to the human Werner/Bloom Syndrome protein), and two partially redundant Dicer proteins (DCL-1 and DCL- 2) are required for silencing [7],[17]–[21]. QDE-1 and QDE-3 are thought to be involved in the generation of dsRNA. It has been proposed that the introduction of a transgene leads to the production of transgene-specific aRNA transcripts, which are then specifically recognized and converted to dsRNA by QDE-1 [2],[22]. The mechanism of aRNA production and the role of QDE-3 in this process are not known. Subsequently, Dicers cleave the dsRNA into siRNA duplexes of around 25 nt which are loaded onto QDE-2 and processed to single-stranded siRNAs with the assistance of the QIP exonuclease, resulting in the activation of RISC [19],[21].

We previously discovered that dsRNA, but not siRNA, activates the transcription of *qde-2*, as well as other RNAi and putative antiviral genes [23]. More recently, we found that DNA damage induces the expression of the Argonaute protein QDE-2 and a class of small RNAs named qiRNAs for their association with QDE-2 [24]. qiRNAs originate mostly from the ribosomal DNA (rDNA) locus, the only highly repetitive DNA locus in *Neurospora*, and their production depends on QDE-1, QDE-3, and Dicers. qiRNA biogenesis also requires the DNA damage-induced aRNA from the rDNA locus. In *qde-1* and *qde-3* mutants, the induction of rDNA-specific aRNA by DNA damage is abolished, indicating their essential roles in aRNA production. Surprisingly, partially purified RdRP QDE-1 can generate RNA from single-stranded DNA (ssDNA) in vitro, suggesting that QDE-1 is also a DdRP that generates aRNA and then converts it into dsRNA using its RdRP activity.

In this study, we demonstrate that QDE-1 is indeed a bona fide DNA-dependent RNA polymerase: recombinant QDE-1 displays DdRP activity that is much more robust than its RdRP activity. In addition, we further investigate the mechanism of aRNA and dsRNA production after DNA damage. Our genetic and biochemical results support a model in which QDE-1 is recruited by ssDNA-binding protein Replication Protein A (RPA) and the RecQ DNA helicase QDE-3. QDE-1 first acts as a DdRP to produce ssRNA and then as an RdRP to convert the ssRNA into dsRNA, a process that is strongly promoted by RPA. These results suggest a mechanism for the generation of aRNA and provide a potential explanation for how aRNA is specifically recognized by RdRPs.

## Results

### Biochemical Analyses of QDE-1 RdRP and DdRP Activities

**Figure 1:**
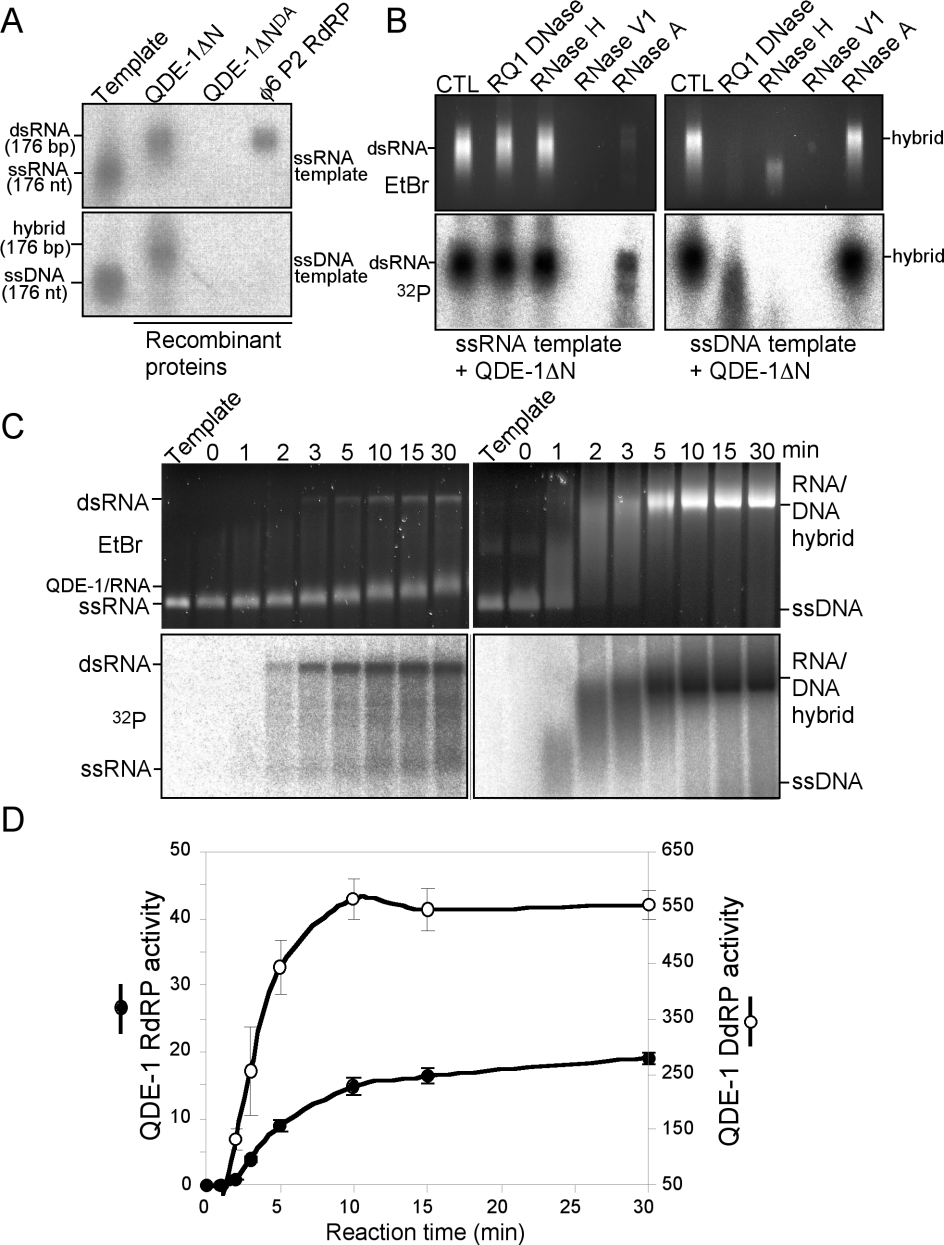
Recombinant QDE-1 exhibits both RdRP and DdRP activities. (A) In vitro RNA polymerase assay using the 176 nt ssDNA or ssRNA templates and recombinant QDE-1ΔN, catalytically inactive QDE-1ΔN^DA^ or bacteriophage φ6 RdRP. ^32^P-labeled reaction products were resolved on a 3% TBE agarose gel. The template lanes contain the corresponding 5′-terminally ^32^P-labeled ssDNA or ssRNA templates. In the other lanes, the templates were not labeled. (B) Characterization of the QDE-1ΔN products from DdRP and RdRP assays by various nuclease treatments. The upper panels show images of ethidium bromide (EtBr) stained gels, and the lower panels are autoradiographs of the same gels. CTL, control. (C and D) In vitro polymerase assays using QDE-1ΔN and a 3 kb ssRNA or ssDNA template. In (C), both ethidium bromide (EtBr) stained images and autoradiographs of the gels are shown. The reactions were initiated by the addition of QDE-1ΔN and samples were collected at the indicated time points. In (D), the values in arbitrary units of incorporated radioactivity were quantified for each product from three independent experiments. The error bars indicate standard errors of the mean. Note the different activity scales used for the ssDNA- or ssRNA-templated assays.

The crystal structure of QDE-1 has shown that its catalytic core is structurally similar to eukaryotic DNA-dependent RNA polymerases [25]. We previously showed that partially purified QDE-1 from *Neurospora* exhibits both RdRP and DdRP activities [24]. To rule out the possibility that another QDE-1-associated polymerase is responsible for this DdRP activity and to biochemically characterize the enzymatic activities of QDE-1, we purified the recombinant catalytically active C-terminal portion of QDE-1 (QDE-1ΔN, residues 377–1402) or the full-length QDE-1 expressed in *Saccharomyces cerevisiae* to near homogeneity (Figure S1) [8]. Both the full-length and truncated forms of QDE-1 exhibited similar activities in our biochemical assays (Figure S2), but due to the ease of expressing QDE-1ΔN in yeast, it was used in most of the assays described in this study. We designed a synthetic 176 nt ssDNA oligonucleotide corresponding to a region of enhanced green fluorescent protein sequence. An ssRNA of the same length and sequence was used as the control template. As shown in Figure 1A (native agarose gel), QDE-1ΔN can use both ssRNA and ssDNA as templates to synthesize radioactively labeled products in a reaction mixture containing all four ribonucleotides and α-^32^P-UTP. In contrast, a recombinant RdRP of the bacteriophage φ6 can only use ssRNA as its template. In addition, the DdRP and RdRP activities of QDE-1ΔN were completely abolished when its conserved catalytic aspartatic acid residue (D1011) was mutated to alanine (QDE-1ΔN^DA^) [8], suggesting that the same catalytic core of QDE-1 is responsible for both DdRP and RdRP activities.

To determine the nature of the ssDNA-templated reaction products by QDE-1, we subjected them to several different nuclease treatments (Figure 1B, right panels). RNase H, which cleaves the RNA strand of a DNA/RNA hybrid, degraded the ^32^P-labeled RNA products and shifted the ethidium bromide stained DNA band to template length. RQ1 DNase, which degrades DNA, shifted the majority of the labeled RNA products to the template length. RNase V1, which cleaves base-paired DNA or RNA, degraded the product completely. In contrast, RNase A, which degrades RNA with single-stranded character, did not affect the reaction products. These results indicate that the products of DdRP activity of QDE-1 are mostly ssRNA hybridized to its template as DNA/RNA hybrids. As expected, the RdRP products of QDE-1ΔN were dsRNA, as neither RQ1 DNase nor RNase H had any effect on the products, while RNase V1 treatment resulted in their complete degradation (Figure 1B, left panels). RNase A treatment resulted in partial degradation of the dsRNA. This is due to the small single-stranded stretch of RNA that bridges the two strands together and results from RNA synthesis initiation mode known as back-priming (see below) [8].

To compare the DdRP and RdRP activities of QDE-1, we used 3 kb ssDNA and ssRNA templates of the same length and sequence in RNA polymerase assays. As shown in Figure 1C (native agarose gels), in the presence of the ssDNA template, QDE-1ΔN rapidly converted almost all DNA templates into DNA/RNA hybrids within 5 min, as indicated by the disappearance of the ssDNA template and the appearance of a strong high molecular weight DNA/RNA hybrid band (lower panels). In contrast, when ssRNA was used as the template, most of the templates remained single-stranded after 30 min of the reaction and were not in dsRNA form (upper panels). The slight mobility change of ssRNA templates seen in the ethidium bromide-stained gel after the reaction was due to the formation of the QDE-1/ssRNA complex and not due to the RNA polymerase reaction because such a mobility shift was still observed in the absence of NTP and could be removed by proteinase K treatment (Figure S3A). Comparison of the two activities showed that the DdRP activity of QDE-1 was about 25-fold higher than its RdRP activity (Figure 1D). These results demonstrate that QDE-1 is a robust DNA-dependent RNA polymerase. Similar results were also obtained with the full-length QDE-1 (Figure S2).

Furthermore, QDE-1ΔN cannot bind dsDNA and was inactive when dsDNA was used as the template (unpublished data). Similarly, Myc-QDE-1 purified from *Neurospora* exhibited no RNA polymerase activity with dsDNA (Figure S3B).

### RPA, an ssDNA-Binding Protein Complex That Interacts with QDE-1, Is Required for the Generation of aRNA and qiRNA

If QDE-1 functions as the RNA polymerase that generates the aberrant transcripts in vivo, it needs to be recruited to ssDNA where the aRNAs are expressed. Previously, QDE-1 was found to interact with Replication Protein A (RPA) [26], but the role of RPA in gene silencing is not known. RPA is an ssDNA binding protein complex and is a major component involved in DNA replication, repair, and recombination pathways [27],[28]. It is a conserved eukaryotic heterotrimeric complex, consisting of RPA1 (RPA70 in mammals), RPA2 (RPA32), and RPA3 (RPA14), that prevents ssDNA from damage, secondary structure formation, and re-annealing in DNA processing pathways.

**Figure 2:**
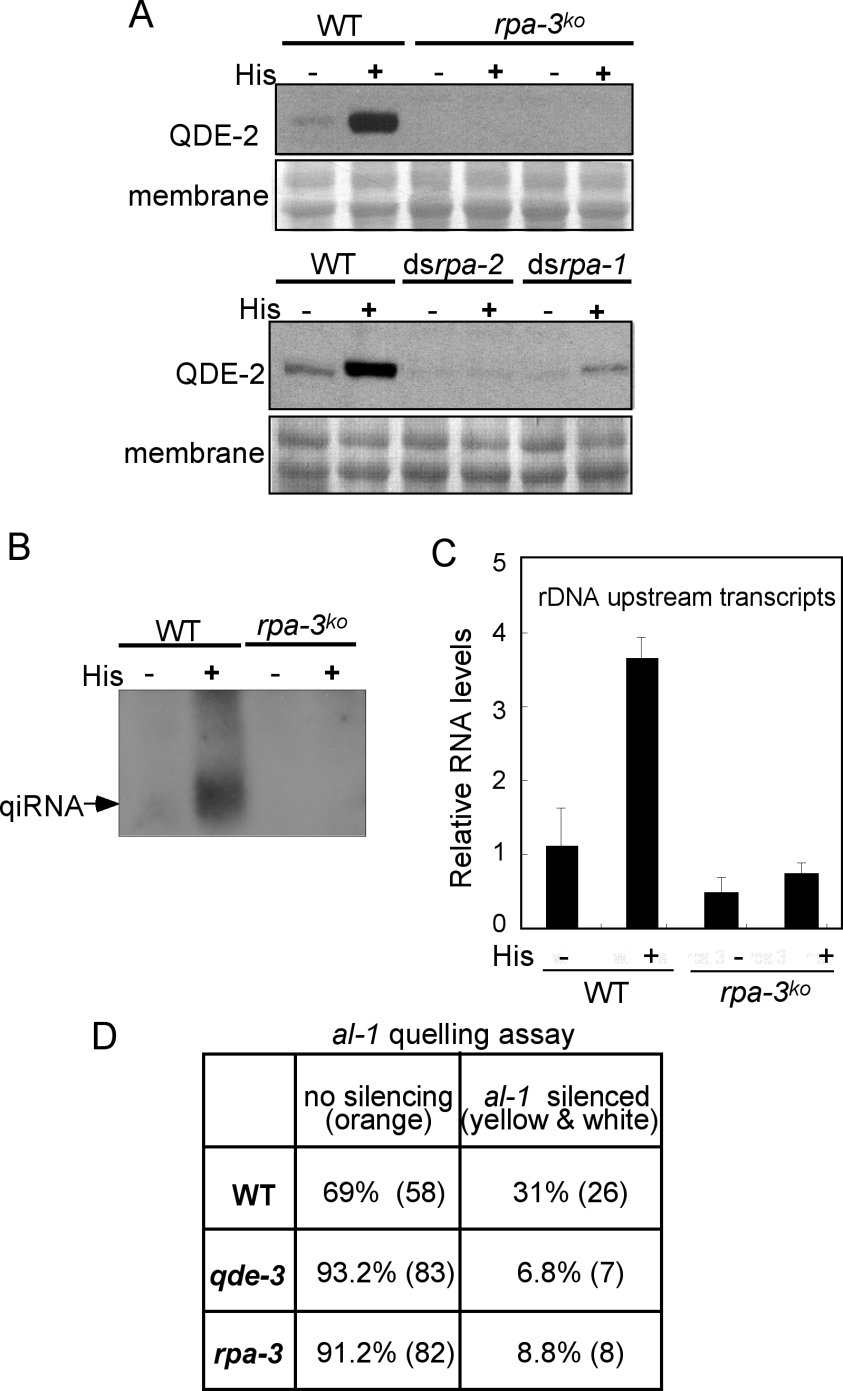
RPA is required for aRNA and qiRNA production and for quelling. (A) Western blot analyses of QDE-2 induction by histidine in the wild type (WT), *rpa-3* knock-out (*rpa-3^ko^*), *rpa-1* knock-down (ds*rpa-1*), and *rpa-2* knock-down (ds*rpa-2*) strains. Two independent *rpa-3^ko^* strains were used. (B) Northern blot analysis of qiRNA production in the wild type (WT) and *rpa-3^ ko^* strains after histidine treatment. Histidine induces DNA damage in *Neurospora*. (C) qRT-PCR showing the production of DNA damage-induced aRNA from the rDNA locus after histidine treatment in wild type and *rpa-3^ko^* strains. (D) The results of the quelling assay showing the silencing efficiency of *albino-1 (al-1)* gene in the wild-type, *qde-3^ko^*, and *rpa-3^ko^* strains. Percentage and actual numbers (shown in parenthesis) of *al-1* silenced transformants are shown.

The QDE-1-RPA interaction suggests that RPA may be the protein that recruits QDE-1 to ssDNA. If so, RPA should be required for the production of aRNA and qiRNA. To test this hypothesis, we sought to generate *rpa* mutants in *Neurospora*. The knock-outs of *rpa-1* and *rpa-2* are lethal, but the knock-out of *rpa-3*, the smallest subunit of RPA, is viable and does not cause severe growth and developmental defects. To obtain *rpa-1* and *rpa-2* knock-down strains, we introduced constructs that can express an inverted repeat specific for *rpa-1* (ds*rpa-1*) or *rpa-2* (ds*rpa-2*) into a wild-type strain. We first measured the induction of QDE-2 by DNA damage agent histidine, an indication of endogenous dsRNA generation [23],[24]. As shown in Figure 2A, the induction of QDE-2 was completely abolished in two independent *rpa-3^ko^* mutants, and the basal levels of QDE-2 in the *rpa-3^ko^* mutants were lower than the basal wild-type level. Also, in the ds*rpa-1* and ds*rpa-2* knockdown strains, the induction of QDE-2 by histidine was mostly abolished. In addition, DNA damage-induced qiRNA and aRNA expression at the rDNA region were both eliminated in the *rpa-3^ko^* mutant (Figure 2B and 2C). Therefore, like QDE-1 and QDE-3, RPA is required for the generation of qiRNA and the rDNA-specific aRNAs.

### RPA Is Required for Quelling

In addition to their roles in qiRNA production, QDE-1 and QDE-3 are also important components in the quelling pathway. The similarity between the qiRNA biogenesis pathway and the quelling pathway prompted us to examine the role of RPA in quelling by transforming a truncated *al-1* gene into the *rpa-3^ko^* and *qde-3^ko^* strains. As shown in Figure 2D, the quelling efficiency of the *rpa-3^ko^* strain was significantly lower than that of the wild-type strain and was comparable to that of the *qde-3^ko^* strain. Both *qde-1* and *qde-3* strains exhibit low levels of quelling but are not completely deficient using our quelling protocol, most likely due to the generation of inverted repeats after transformation, which can allow the generation of dsRNA, bypassing the requirement of QDE-1 and QDE-3 [18],[24]. On the other hand, the *qde-2* and the *dcl-1/dcl-2* double mutant strains are completely deficient in quelling [29]. These results indicate that, like QDE-1 and QDE-3, RPA is an important component in the quelling pathway.

### QDE-1 Can Initiate RNA Synthesis Internally from ssDNA Templates and Can Produce dsRNA Directly from ssDNA

**Figure 3:**
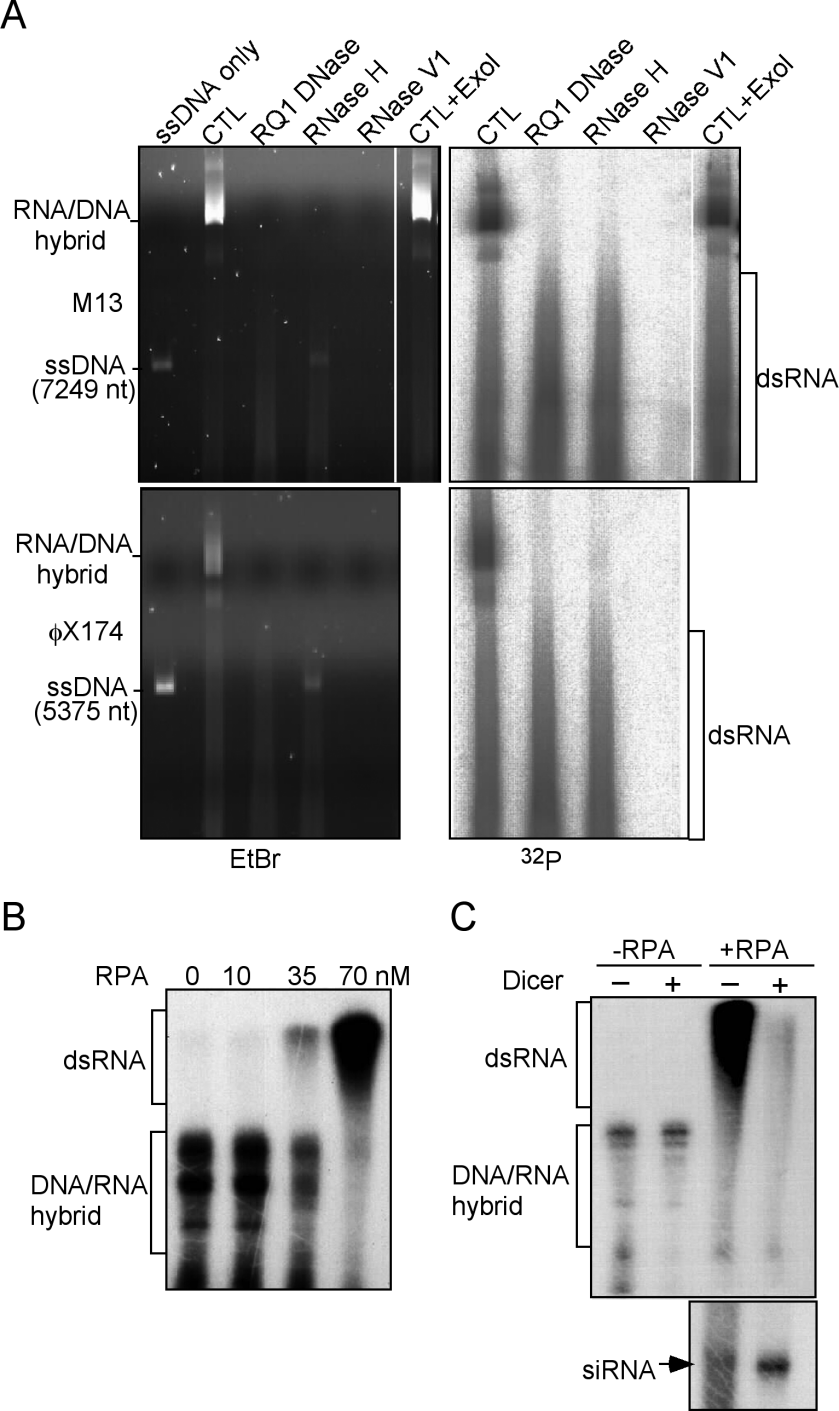
RPA promotes ssDNA-directed synthesis of dsRNA by QDE-1. (A) In vitro DdRP assay using circular ssDNA from bacteriophages M13 or φX174 as templates. The nature of the products was characterized by various nuclease treatments and native agarose gel (0.6%) electrophoresis. (B) In vitro DdRP assay using full-length recombinant QDE-1 with various concentrations of the RPA complex. The 175 nt ssDNA template was pre-incubated with RPA before adding QDE-1, and the products were resolved by 6% urea containing polyacrylamide gel. The DNA/RNA hybrid refers to the single-stranded, labeled RNA products of the denatured hybrids. The dsRNA species migrated at higher molecular weight positions in the denaturing gels due to their synthesis by back-priming initiation. (C) The QDE-1 RdRP products with 0 or 70 nM RPA were treated with recombinant Dicer and resolved in 6% (top) or 16% (bottom) urea-containing polyacrylamide gels. The DNA/RNA hybrid refers to the single-stranded, labeled RNA products of the denatured hybrids.

Since free ssDNA ends are rare in vivo and mostly protected by proteins, we examined whether QDE-1 can initiate RNA synthesis internally from ssDNA. In addition, since QDE-1 generates ssRNA from ssDNA, we wanted to know whether QDE-1 can directly use the ssRNAs it produces as templates to generate dsRNA. To test these possibilities, we performed RNA polymerase assays using long circular ssDNA templates (genomic ssDNA of bacteriophage M13mp18 or φX174, 7.2 and 5.4 kb, respectively). As shown in Figure 3A (native agarose gels), QDE-1ΔN exhibited robust DdRP activity with both circular templates and the band corresponding to ssDNA templates disappeared (the CTL lanes). In addition, treatment of M13 ssDNA template with Exonuclease I, which degrades linear ssDNA prior to the reactions, did not affect the activity of QDE-1. These results indicate that QDE-1 can indeed initiate RNA synthesis internally from ssDNA templates.

There are two types of products made by QDE-1 using these circular ssDNA templates as indicated by the prominent high molecular weight bands and the low molecular weight smear (Figure 3A). The primary high molecular weight QDE-1 products were degraded by both RQ1 DNase and RNase H. Furthermore, the RNase H treatment resulted in the reappearance of the ssDNA template band, indicating that the main products of the QDE-1 catalyzed reaction are DNA/RNA hybrids. In contrast, the low molecular weight smear products were resistant to both RQ1 DNase and RNase H but were completely degraded by RNase V1. Since RNase V1 degrades base-paired RNA or DNA, these results showed that the low molecular weight smear products are mostly dsRNA. These data also indicate that some of the short nascent RNAs can dissociate from ssDNA, providing free ssRNA for dsRNA synthesis by QDE-1, resulting in dsRNA products of variable lengths. Thus, QDE-1 can directly generate dsRNA from ssDNA templates.

### RPA Strongly Promotes the Ability of QDE-1 to Produce dsRNA by Preventing the Formation of DNA/RNA Hybrids

Although QDE-1 can produce some dsRNA from ssDNA, the majority of the QDE-1 products are DNA/RNA hybrids, which prevent the generation of dsRNA. Thus, for robust dsRNA production, a mechanism should exist to unwind the nascent DNA/RNA hybrids or to prevent their formation. Since RPA binds to ssDNA, we examined its effect on QDE-1 RNA polymerase activity. In addition, we were interested to know whether QDE-1 can use RPA-bound ssDNA as a template. Different concentrations of human RPA complex were incubated with ssDNA (a 175 nt template) before the addition of recombinant full-length QDE-1. The reaction products were separated by a denaturing polyacrylamide gel. As shown in Figure 3B and 3C, without RPA or at a low RPA concentration (10 nM), the QDE-1 products were mostly DNA/RNA hybrids. However, when RPA concentration was increased to 35 nM, the DNA/RNA hybrid bands decreased and a high molecular weight band appeared. At 70 nM of RPA, the DNA/RNA hybrid bands completely disappeared and the high molecular weight forms became the only major QDE-1 products. This result demonstrates that QDE-1 can use RPA-bound ssDNA as a template and RPA can regulate the RNA polymerase activity of QDE-1. We also performed polymerization reactions with QDE-1 and the single-stranded DNA binding protein (SSB) of *E. coli* but failed to detect a clear effect on QDE-1 activity (unpublished data). This suggests that the effect of RPA on QDE-1 activity extends beyond its ssDNA binding character.

It was previously shown that for ssRNA templates in vitro, QDE-1 can utilize the 3′ ends of ssRNAs to create dsRNAs in which the complementary strands are covalently attached to each other, as a result of a mode of initiation called back-priming [8]. The high molecular weight products detected in the denaturing polyacrylamide gel suggest that they are most likely dsRNA molecules synthesized by back-priming initiation. To confirm this, the QDE-1 products synthesized in the presence or absence of RPA were subjected to Dicer treatment (recombinant *Drosophila* Dicer-2 purified from Sf9 insect cells). As shown in Figure 3C, Dicer treatment removed the vast majority of the high molecular weight QDE-1 products but the DNA/RNA hybrids were unaffected. Note that some of the small QDE-1 products made in the absence of RPA were also removed by Dicer. Subsequently, the Dicer-treated products were separated in a 16% polyacrylamide gel to detect the presence of siRNAs. As shown in Figure 3C (bottom panel), the QDE-1 products synthesized in the presence of RPA resulted in a significant increase of siRNA level after the Dicer treatment. Together, these results suggest that RPA strongly promotes the ability of QDE-1 to synthesize dsRNA from ssDNA by preventing the formation of DNA/RNA hybrids.

### The Interaction between QDE-1 and RPA Requires QDE-3

RPA participates in many DNA metabolic processes under normal growth conditions, but qiRNA and rDNA-specific aRNA are preferentially produced after treatments with DNA damaging agents, suggesting that the interaction between QDE-1 and RPA requires additional determinant(s). QDE-3 and its eukaryotic homologs such as human BLM and WRN and yeast SGS1 play important roles in DNA repair, genome maintenance, and DNA replication [20],[30]–[33]. These QDE-3 homologs display ATP dependent 3′–5′ DNA helicase activity to unwind duplex DNA and are recruited to damaged replication forks after treatment with DNA damaging agents or a blockade of replication. In addition, it has been shown that RPA interacts with BLM and WRN proteins and that RPA stimulates their DNA helicase activity [33],[34].

**Figure 4:**
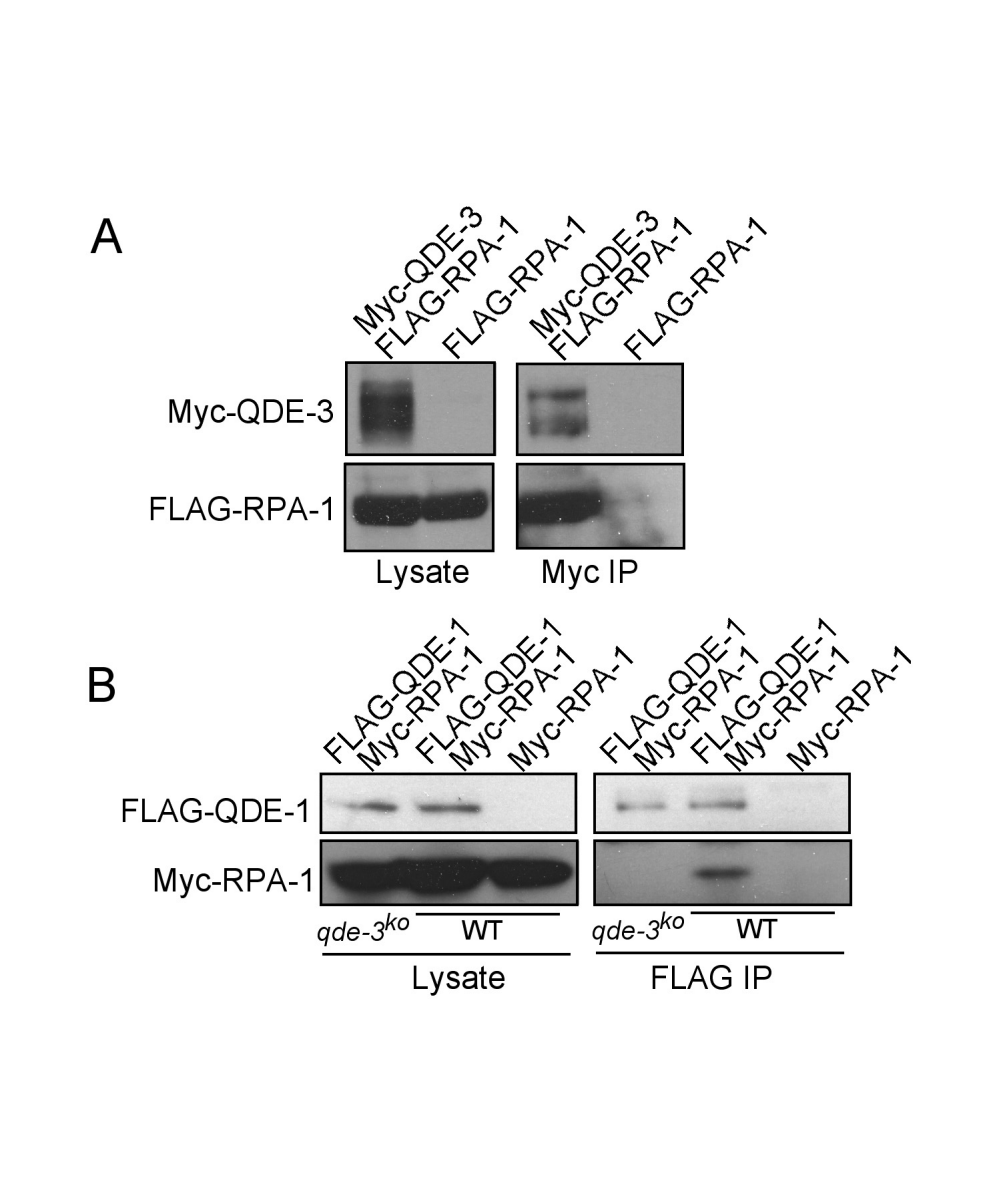
The interaction between QDE-1 and RPA-1 requires QDE-3. c-Myc-tagged QDE-3 and FLAG-tagged RPA-1 constructs were co-transformed into wild-type *Neurospora* (A and B) and *qde-3^ko^* (B) strains. (A) Immunoprecipitation (IP) with a c-Myc specific antibody showing the interaction of c-Myc-QDE-3 with FLAG-RPA-1. The strain expressing only FLAG-RPA-1 was used as the control. (B) FLAG-IP showing the interaction between FLAG-QDE-1 and c-Myc-RPA-1, and the disappearance of this interaction in the *qde-3^ko^* strain. The strain expressing only Myc-RPA-1 was used as the control. The antibodies used in the western analyses are indicated on the left. The strains and the fusion protein expressed are indicated below and above the panels, respectively.

To understand the function of QDE-3 in aRNA production, we examined its interaction with RPA in *Neurospora*. c-Myc-tagged QDE-3 and FLAG-tagged RPA-1 constructs under the control of the *Neurospora* quinic acid inducible promoter (a rather weak promoter) were co-transformed into a wild-type *Neurospora* strain. As shown in Figure 4A, Myc-QDE-3 was found to interact specifically with FLAG-RPA-1 by immunoprecipitation (IP) assay, suggesting that QDE-3 and RPA work together. Consistent with earlier results [26], we found that QDE-1 interacts with RPA-1 in the wild-type *Neurospora* strain (Figure 4B). However, this interaction was abolished in the *qde-3^ko^* strain, indicating that QDE-3 is important for the interaction between QDE-1 and RPA. Since QDE-3 is involved in DNA repair and its homologs are known to be recruited to DNA damage sites, these results suggest that although RPA is involved in normal DNA metabolism, it interacts with QDE-1 when QDE-3 is present at the damaged loci. Thus, aRNA and qiRNA are only induced after treatments with DNA damaging agents.

## Discussion

The genetic and biochemical results presented here and in previous studies [24],[26] suggest that in *Neurospora*, QDE-1 acts both as a DdRP and an RdRP to generate aRNA and the subsequent dsRNA necessary to initiate the RNAi pathway. This conclusion is supported by several lines of evidence. (i) QDE-1, but not the canonical RNA polymerases, is required for aRNA production from the rDNA locus after treatment with DNA damaging agents [24]. (ii) Both the recombinant QDE-1 and QDE-1 purified from *Neurospora* exhibit robust DdRP activity using ssDNA but not dsDNA templates, and the DdRP activity of QDE-1 is considerably higher than its RdRP activity. (iii) QDE-1 can directly generate dsRNA from ssDNA templates. (iv) RPA, a QDE-1-interacting protein that binds to ssDNA, is required for aRNA and qiRNA production and strongly enhances dsRNA synthesis. Therefore, although we cannot exclude the possibility that other RNA polymerases also contribute to the rDNA specific aRNA production, the evidence suggests that QDE-1 should be a major RNA polymerase involved in this process. Although it was previously shown that QDE-1 can produce some small RNAs from ssRNA in vitro [8], this activity does not appear to be relevant in vivo since QDE-1 is not required once dsRNA is made and is not involved in making secondary small RNAs [18],[24].

Our results suggest that the generation of ssDNA after DNA damage/replication stress is a trigger for the production of aRNA and small RNA. The requirement of RPA in aRNA and qiRNA production and the interaction between RPA and QDE-1 support this model and suggest that QDE-1 may be recruited to ssDNA template by RPA. In addition, like their mammalian homologs, RPA and QDE-3 apparently associate with each other in vivo (Figure 4). Since RPA can stimulate the DNA helicase activity of QDE-3 homologs in mammals [33],[34], it is plausible that in *Neurospora*, QDE-3 and RPA are responsible for the generation of ssDNA template for QDE-1. Furthermore, the loss of QDE-1-RPA interaction in the *qde-3^ko^* mutant suggests that QDE-3 contributes to this interaction. The involvement of QDE-3 in this process suggests that although RPA is involved in many processes of DNA metabolism and ssDNA is generated during normal DNA replication, QDE-1 is only recruited to the ssDNA locus if both RPA and QDE-3 are present. This conclusion is supported by the observation that qiRNA and aRNA production is abolished in the *qde-3^ko^* mutant [24].

Our results also provide a molecular explanation for how aRNAs but not other cellular RNAs are specifically recognized by QDE-1. In plants and yeast, it was proposed that RdRPs recognize their specific templates through the recruitment of RdRP by Argonaute-siRNA complexes [1]. However, QDE-2 Argonaute is not required for the production of aRNA and qiRNA, suggesting a different mechanism of aRNA recognition by QDE-1 [24]. The results presented here suggest that since the aRNA is generated by QDE-1, the close proximity between the two allows it to use the nascent aRNA as a template to make dsRNA. Thus, there is no need for a mechanism to specifically recruit QDE-1 to aRNA. Notably, the recombinant QDE-1 forms a homodimer [25], and thus it may employ one of its active sites for aRNA synthesis and have the other one on “standby” to convert the nascent ssRNA into dsRNA. Therefore, the specificity between QDE-1 and aRNA is determined by their shared location and by the biochemical activity of QDE-1.

Although most of the in vitro DdRP products of QDE-1 are DNA/RNA hybrids, some free ssRNA can be generated, as indicated by the generation of some dsRNA from ssDNA. Importantly, we showed that RPA strongly promotes dsRNA production by QDE-1 from ssDNA by preventing the formation of DNA/RNA hybrids. Therefore, RPA may have two roles in this small RNA production process: recruiting QDE-1 to ssDNA and blocking the formation of DNA/RNA hybrids. It has been shown that the human QDE-3 homolog BLM can unwind DNA/RNA hybrids to release free ssRNAs [35]. Thus, QDE-3 may also potentially contribute to the unwinding of the RNA/DNA hybrid in vivo.

Although we use the DNA damage-induced aRNA as a model, the mechanism of aRNA production is most likely shared by quelling, a post-transcriptional silencing mechanism against repetitive transgenes. We showed that, like QDE-1 and QDE-3, RPA is also required for quelling. In addition, QDE-1 is recruited to the transgenic locus upon quelling [26], consistent with the model we proposed here. However, QDE-1 and QDE-3 are not required for the production of other types of small RNAs, including miRNA-like RNAs and Dicer-independent small RNAs in *Neurospora* [36].

The rDNA region is the only highly repetitive DNA locus in the wild-type *Neurospora* genome. In the case of quelling, which is triggered by the transformation of transgene DNA, a second highly repetitive DNA locus with tandem repeats of transgene is created. Thus, the repetitive nature of the rDNA and the quelled locus is likely to be the cause for aRNA and small RNA production. In *Neurospora*, the rDNA region is a known site of frequent chromosome breakage [37]. It is possible that aberrant DNA structures at the repetitive DNA loci due to mutation or recombination can result in frequent replication fork stall (enhanced by DNA damage agent treatment), which may recruit QDE-3, RPA, and QDE-1 to these loci to produce ssDNA and then aRNA and dsRNA.

In *Arabidopsis*, the partially purified RdRP RDR6, which plays an important role in RNAi pathways, has also been shown to have a strong DdRP activity on ssDNA and during dsRNA synthesis, but it cannot distinguish between template RNAs with or without a cap or poly(A) tail in vitro [38]. Furthermore, a mutation of RPA2 in *Arabidopsis* impairs transcriptional gene silencing at certain loci [39]. These results suggest that RdRPs and RPA may have similar roles in plants as in *Neurospora*, although it is possible that they may function differently from those in *Neurospora*. Interestingly, piRNAs from rat testes were found to be associated with rRecQ1 [40], a QDE-3 homolog, raising the possibility that rRecQ1 may have a role in generating primary transcripts, which can be used as the precursor for piRNAs in mammals.

## Materials and Methods

### Strains and Culture Conditions

A wild type strain of *Neurospora crassa* (FGSC4200) was used in this study unless otherwise mentioned. The *rpa-3* (NCU01460.3) knock-out strain was obtained from Fungal Genetic Stock Center (FGSC) [41]. The knock-down strains of ds*rpa-1* (NCU03606.3) and ds*rpa-2* (NCU07717.3) were created as previously described [42]. Transgenic strains expressing c-Myc or FLAG tagged proteins were created by plasmid transformation into *his-3* locus by electroporation. Strains co-expressing both c-Myc and FLAG tagged proteins were generated by cotransformation of plasmid containing the FLAG tagged gene with plasmid (pBT6) containing benomyl-resistance into the strains expressing the c-Myc-tag protein.

For the expression of inverted repeats and tagged proteins in *Neurospora*, 0.01 M QA (pH 5.8) was added to the liquid culture medium containing 1×Vogel's, 0.1% glucose, and 0.17% arginine. DNA damage was induced by the addition of histidine (100 µg/ml), and samples were harvested 40 h later [24].

### Plasmid Construction

A FLAG (3×FLAG) tag containing plasmid was a kind gift from Dr. James Chen at University of Texas Southwestern Medical Center. The FLAG tag was subcloned downstream of the *qa-2* promoter, creating plasmid qa-3FLAG. Full-length *qde-1* or *rpa-1* genes were then inserted in frame following the FLAG tag. To create c-Myc-tagged proteins, full-length genes of *qde-1*, *qde-3*, or *rpa-1* were inserted into a c-Myc tag containing plasmid [43]. Both FLAG and Myc containing plasmids also contain sequences encoding six histidines.

### RNA Analyses

Total RNA extraction, enrichment of low molecular weight small RNA, and Northern blot were performed as previously described [21]. RNA probes were made by MAXIscript T7 kit (Ambion) using T7 promoter containing aPCR products as templates. Quantitative real time PCR experiments were performed as previously described [23]. Specific primer pairs were used to detect intergenic transcripts from the rDNA region (upstream of rRNA coding region) [24].

### Expression and Purification of RdRPs

Recombinant full-length QDE-1, QDE-1ΔN, QDE-1ΔN^DA^, and RdRP of bacteriophage φ6 were expressed and purified as previously described [44],[45]. pEM41, pEM69, and pEM56 expressing full-length QDE-1, QDE-1ΔN, and QDE-1ΔN^DA^, respectively, each with a carboxy terminal 6-His tag, were introduced into *Saccharomyces cerevisiae* strain INVSc1 (Invitrogen). The recombinant proteins were expressed at +28°C for 22 h and purified to near homogeneity. Wild-type recombinant φ6 P2 was expressed in *Escherichia coli* BL21(DE3) strain (Novagen) containing the plasmid pEMG2 [46] at +20°C for 15 h and purified to near homogeneity. The purified proteins were stored in 50 mM Tris-HCl (pH 8.0), 0.1 mM EDTA, 0.13% Triton X-100, 100 mM NaCl, and 62.5% glycerol at −20°C. The full-length 6His tagged c-Myc-His-QDE-1 from *Neurospora* was partially purified by Ni-NTA matrices (QIAGEN) as previously described [24].

### Template RNAs and DNAs

The single-stranded 176 nt DNA oligonucleotide was purchased from biomers.net, and its sequence corresponds to nts 726–901 of pEGFP-C1 (Clontech). For the synthesis of ssRNA of the same sequence, the 176 nt ssDNA was PCR-amplified. The resulting PCR product was purified and used as a template for T7 transcription reaction. The DNA template was then degraded with DNaseI (Promega), and the ssRNA was gel purified. To prepare the 3 kb ssRNA, plasmid pLM659 [47] was linearized by *SmaI* digestion and used as a template for run-off transcription by T7 RNA polymerase. To generate the corresponding ssDNA molecule, pLM659 was used as a template in PCR reactions where one of the primers contained a 5′-biotin. The biotinylated PCR product was immobilized onto Dynabeads MyOne Streptavidin C1 magnetic beads (Invitrogen) according to manufacturer's instructions. The immobilized PCR product was dissolved by treating the DNA briefly with fresh 0.1 M NaOH. The ssDNA was subsequently precipitated and gel purified. For some experiments (Figures S2B, 3B and 3C), the 175 nt ssDNA, deriving from mature 26S rRNA region, was made by boiling followed by rapid chilling on ice water [24]. The genomic ssDNAs of bacteriophages φX174 and M13mp18 were purchased from New England Biolabs.

### RNA Polymerase Assays

RNA polymerase reactions were performed as described [8],[45]. The reactions were carried out at 30°C for 60 min unless otherwise noted. Reaction products were subjected to gel electrophoresis using native agarose gel or denaturing polyacrylamide (6% or 16%) TBE gels. Radioactivity was detected by phosphorimaging and analyzed by densitometry with AIDA software (Raytest Isotopenmessgerate GmbH). For nuclease assays of reaction products, RNasin Ribonuclease Inhibitor (Promega) was omitted from the reactions, and the reaction products were extracted with phenol:chloroform, precipitated with NH4OAc and ethanol, and dissolved in water. Subsequently, equal amounts of reaction products were supplemented with 0.1 U/µl of RQ1 DNase (Promega), 0.5 U/µl of RNase H (Fermentas), 0.01 U/µl of RNase V1 (Ambion), or 0.1 ng/µl RNase A (Ambion), and their respective 1× reaction buffers, incubated for 30 min at +37°C and analyzed by electrophoresis. For reactions with RPA, the ssDNA template was first incubated with RPA at +37°C for 10 min before adding recombinant QDE-1. Recombinant human RPA heterotrimer is a generous gift from Dr. Marc Wold [48].

### Protein Analysis

Protein extraction, quantification, immunoprecipitation (IP), and Western blot analysis were performed as previously described [21],[49]. Equal amounts of total protein (50 µg) were separated in SDS-PAGE and transferred onto PVDF membrane. For Western blot, a monoclonal c-Myc antibody (Roche 9E10) and anti-FLAG M2 antibody (Sigma) were used. For immunoprecipitation, anti-c-Myc and anti-FLAG antibodies were used at 1∶1000 and 1∶300 dilutions, respectively.

### Quelling Assay

*Neurospora* strains were co-transformed with 0.5 µg pBSK*al-1* plasmid (contains a truncated *abino-1 (al-1)* gene) and 0.5 µg pBT6 plasmid. The benomyl-resistant colonies were picked and grown on slants. The colors of around 100 transformants from each strain were observed for comparing silencing (quelling) efficiency of the *al-1* gene.

## Supporting Information

Figure S1 **Coomassie-stained SDS-PAGE gels showing the purified truncated (above) and full-length QDE-1 (below).** Recombinant QDE-1 proteins expressed in yeast were purified by a Ni-NTA column, a heparin column followed by an ion-exchange column. Protein fractions after the ion-exchange column are shown. The top fractions were pooled and concentrated before use in RNA polymerase assays. Full-length QDE-1 is ∼160 kDa and QDE-1 ΔN is ∼120 kDa in size.
(0.20 MB PDF)

Figure S2 **Full-length, recombinant QDE-1 was used in the same RNA polymerase assay as described in** **Figure 1C** **, and shown are the ethidium bromide stained native agarose gels.** Upper panel: ssRNA template; lower panel: ssDNA template. The activity of the full-length QDE-1 is identical to that of QDE-1 ΔN.
(1.26 MB PDF)

Figure S3 (A) RNA polymerase reactions showing the association of QDE-1 with ssRNA and ssDNA in the presence and absence of NTP. ^32^P-labeled ssRNA (left panel) and ssDNA (right panel) templates were used. (B) RNA polymerase reactions using Myc-QDE-1 purified from *Neurospora* showing that QDE-1 cannot use dsDNA as a template.
(0.47 MB PDF)

## Abbreviations

aRNA: aberrant RNA
DdRP: DNA-dependent RNA polymerase
ds: double-stranded
qiRNA: QDE-2-interacting small RNA
RdRP: RNA-dependent RNA polymerase
RPA: Replication Protein A
siRNA: small interfering RNA
ss: single-stranded

## References

[1] Buhler M, Moazed D (2007). Transcription and RNAi in heterochromatic gene silencing.. Nat Struct Mol Biol.

[2] Catalanotto C, Nolan T, Cogoni C (2006). Homology effects in Neurospora crassa.. FEMS Microbiol Lett.

[3] Hannon G. J (2002). RNA interference.. Nature.

[4] Meister G, Tuschl T (2004). Mechanisms of gene silencing by double-stranded RNA.. Nature.

[5] Mello C. C, Conte D (2004). Revealing the world of RNA interference.. Nature.

[6] Ghildiyal M, Zamore P. D (2009). Small silencing RNAs: an expanding universe.. Nat Rev Genet.

[7] Cogoni C, Macino G (1999). Gene silencing in Neurospora crassa requires a protein homologous to RNA-dependent RNA polymerase.. Nature.

[8] Makeyev E. V, Bamford D. H (2002). Cellular RNA-dependent RNA polymerase involved in posttranscriptional gene silencing has two distinct activity modes.. Mol Cell.

[9] Sijen T, Fleenor J, Simmer F, Thijssen K. L, Parrish S (2001). On the role of RNA amplification in dsRNA-triggered gene silencing.. Cell.

[10] Xie Z, Johansen L. K, Gustafson A. M, Kasschau K. D, Lellis A. D (2004). Genetic and functional diversification of small RNA pathways in plants.. PLoS Biol.

[11] Pak J, Fire A (2007). Distinct populations of primary and secondary effectors during RNAi in C. elegans.. Science.

[12] Sijen T, Steiner F. A, Thijssen K. L, Plasterk R. H (2007). Secondary siRNAs result from unprimed RNA synthesis and form a distinct class.. Science.

[13] Herr A. J, Jensen M. B, Dalmay T, Baulcombe D. C (2005). RNA polymerase IV directs silencing of endogenous DNA.. Science.

[14] Onodera Y, Haag J. R, Ream T, Nunes P. C, Pontes O (2005). Plant nuclear RNA polymerase IV mediates siRNA and DNA methylation-dependent heterochromatin formation.. Cell.

[15] Djupedal I, Portoso M, Spahr H, Bonilla C, Gustafsson C. M (2005). RNA Pol II subunit Rpb7 promotes centromeric transcription and RNAi-directed chromatin silencing.. Genes Dev.

[16] Kato H, Goto D. B, Martienssen R. A, Urano T, Furukawa K (2005). RNA polymerase II is required for RNAi-dependent heterochromatin assembly.. Science.

[17] Catalanotto C, Azzalin G, Macino G, Cogoni C (2000). Gene silencing in worms and fungi.. Nature.

[18] Catalanotto C, Pallotta M, ReFalo P, Sachs M. S, Vayssie L (2004). Redundancy of the two dicer genes in transgene-induced posttranscriptional gene silencing in Neurospora crassa.. Mol Cell Biol.

[19] Catalanotto C, Azzalin G, Macino G, Cogoni C (2002). Involvement of small RNAs and role of the qde genes in the gene silencing pathway in Neurospora.. Genes Dev.

[20] Cogoni C, Macino G (1999). Posttranscriptional gene silencing in Neurospora by a RecQ DNA helicase.. Science.

[21] Maiti M, Lee H. C, Liu Y (2007). QIP, a putative exonuclease, interacts with the Neurospora Argonaute protein and facilitates conversion of duplex siRNA into single strands.. Genes Dev.

[22] Cogoni C, Irelan J. T, Schumacher M, Schmidhauser T. J, Selker E. U (1996). Transgene silencing of the al-1 gene in vegetative cells of Neurospora is mediated by a cytoplasmic effector and does not depend on DNA-DNA interactions or DNA methylation.. Embo J.

[23] Choudhary S, Lee H. C, Maiti M, He Q, Cheng P (2007). A double-stranded-RNA response program important for RNA interference efficiency.. Mol Cell Biol.

[24] Lee H. C, Chang S. S, Choudhary S, Aalto A. P, Maiti M (2009). qiRNA is a new type of small interfering RNA induced by DNA damage.. Nature.

[25] Salgado P. S, Koivunen M. R, Makeyev E. V, Bamford D. H, Stuart D. I (2006). The structure of an RNAi polymerase links RNA silencing and transcription.. PLoS Biol.

[26] Nolan T, Cecere G, Mancone C, Alonzi T, Tripodi M (2008). The RNA-dependent RNA polymerase essential for post-transcriptional gene silencing in Neurospora crassa interacts with replication protein A.. Nucleic Acids Res.

[27] Fanning E, Klimovich V, Nager A. R (2006). A dynamic model for replication protein A (RPA) function in DNA processing pathways.. Nucleic Acids Res.

[28] Zou Y, Liu Y, Wu X, Shell S. M (2006). Functions of human replication protein A (RPA): from DNA replication to DNA damage and stress responses.. J Cell Physiol.

[29] Maiti M (2007). The mechanism of RNA interference in *Neurospora* [Ph.D.]..

[30] Bachrati C. Z, Hickson I. D (2003). RecQ helicases: suppressors of tumorigenesis and premature aging.. Biochem J.

[31] Kato A, Akamatsu Y, Sakuraba Y, Inoue H (2004). The Neurospora crassa mus-19 gene is identical to the qde-3 gene, which encodes a RecQ homologue and is involved in recombination repair and postreplication repair.. Curr Genet.

[32] Wu L, Hickson I. D (2006). DNA helicases required for homologous recombination and repair of damaged replication forks.. Annu Rev Genet.

[33] Bachrati C. Z, Hickson I. D (2008). RecQ helicases: guardian angels of the DNA replication fork.. Chromosoma.

[34] Choudhary S, Doherty K. M, Handy C. J, Sayer J. M, Yagi H (2006). Inhibition of Werner syndrome helicase activity by benzo[a]pyrene diol epoxide adducts can be overcome by replication protein A.. J Biol Chem.

[35] Popuri V, Bachrati C. Z, Muzzolini L, Mosedale G, Costantini S (2008). The human RecQ helicases, BLM and RECQ1, display distinct DNA substrate specificities.. J Biol Chem.

[36] Lee H. C, Li L, Gu W, Xue Z, Crosthwaite S. K (2010). Diverse pathways generate MicroRNA-like RNAs and Dicer-independent small interfering RNAs in fungi.. Mol Cell.

[37] Butler D. K (1992). Ribosomal DNA is a site of chromosome breakage in aneuploid strains of Neurospora.. Genetics.

[38] Curaba J, Chen X (2008). Biochemical activities of Arabidopsis RNA-dependent RNA polymerase 6.. J Biol Chem.

[39] Kapoor A, Agarwal M, Andreucci A, Zheng X, Gong Z (2005). Mutations in a conserved replication protein suppress transcriptional gene silencing in a DNA-methylation-independent manner in Arabidopsis.. Curr Biol.

[40] Lau N. C, Seto A. G, Kim J, Kuramochi-Miyagawa S, Nakano T (2006). Characterization of the piRNA complex from rat testes.. Science.

[41] Colot H. V, Park G, Turner G. E, Ringelberg C, Crew C. M (2006). A high-throughput gene knockout procedure for Neurospora reveals functions for multiple transcription factors.. Proc Natl Acad Sci U S A.

[42] Cheng P, He Q, He Q, Wang L, Liu Y (2005). Regulation of the Neurospora circadian clock by an RNA helicase.. Genes Dev.

[43] He Q, Cheng P, He Q, Liu Y (2005). The COP9 signalosome regulates the Neurospora circadian clock by controlling the stability of the SCFFWD-1 complex.. Genes & Dev.

[44] Laurila M. R, Salgado P. S, Makeyev E. V, Nettelship J, Stuart D. I (2005). Gene silencing pathway RNA-dependent RNA polymerase of Neurospora crassa: yeast expression and crystallization of selenomethionated QDE-1 protein.. J Struct Biol.

[45] Makeyev E. V, Bamford D. H (2000). Replicase activity of purified recombinant protein P2 of double-stranded RNA bacteriophage phi6.. Embo J.

[46] Poranen M. M, Koivunen M. R, Bamford D. H (2008). Nontemplated terminal nucleotidyltransferase activity of double-stranded RNA bacteriophage phi6 RNA-dependent RNA polymerase.. J Virol.

[47] Gottlieb P, Strassman J, Qiao X, Frilander M, Frucht A (1992). In vitro packaging and replication of individual genomic segments of bacteriophage phi 6 RNA.. J Virol.

[48] Haring S. J, Mason A. C, Binz S. K, Wold M. S (2008). Cellular functions of human RPA1. Multiple roles of domains in replication, repair, and checkpoints.. J Biol Chem.

[49] Cheng P, Yang Y, Heintzen C, Liu Y (2001). Coiled-coil domain mediated FRQ-FRQ interaction is essential for its circadian clock function in *Neurospora*.. EMBO J.

