# Supplementary figures and images for: The DNA/RNA-Dependent RNA Polymerase QDE-1 Generates Aberrant RNA and dsRNA for RNAi in a Process Requiring Replication Protein A and a DNA Helicase
Source: PLoS Biol. 2010 Oct 5;8(10):e1000496. doi: 10.1371/journal.pbio.1000496 (PMC2950127; doi:10.1371/journal.pbio.1000496)

QDE-1ΔN →

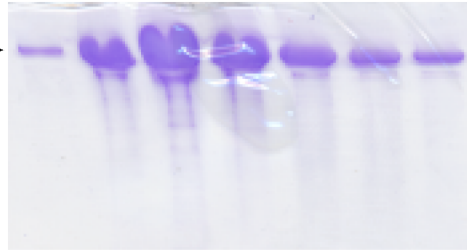

full-length QDE-1 →

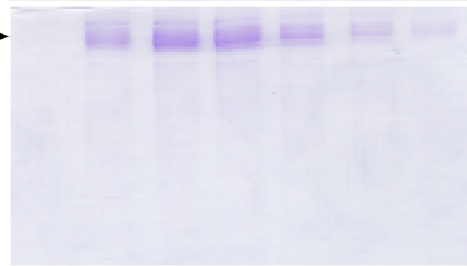

Supplement: Figure S1 — Coomassie-stained SDS-PAGE gels showing the purified truncated (above) and full-length QDE-1 (below). Recombinant QDE-1 proteins expressed in yeast were purified by a Ni-NTA column, a heparin column followed by an ion-exchange column. Protein fractions after the ion-exchange column are shown. The top fractions were pooled and concentrated before use in RNA polymerase assays. Full-length QDE-1 is ∼160 kDa and QDE-1 ΔN is ∼120 kDa in size. (0.20 MB PDF) [file pbio.1000496.s001.pdf]

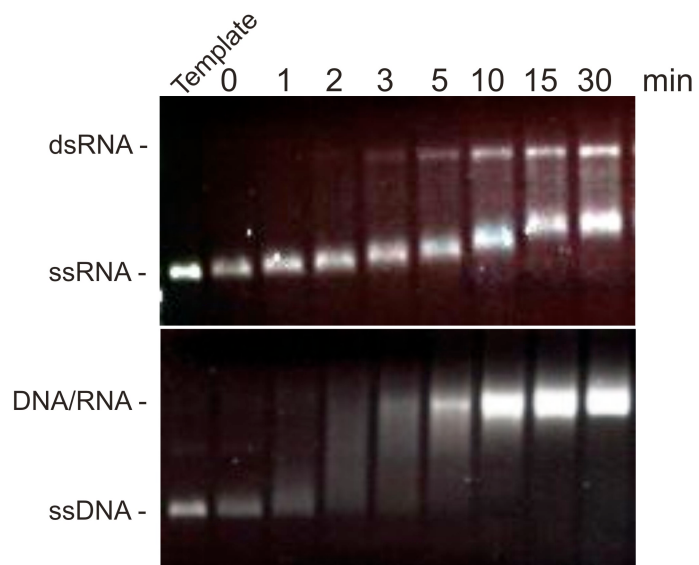

Supplement: Figure S2 — Full-length, recombinant QDE-1 was used in the same RNA polymerase assay as described in Figure 1C , and shown are the ethidium bromide stained native agarose gels. Upper panel: ssRNA template; lower panel: ssDNA template. The activity of the full-length QDE-1 is identical to that of QDE-1 ΔN. (1.26 MB PDF) [file pbio.1000496.s002.pdf]

**A**

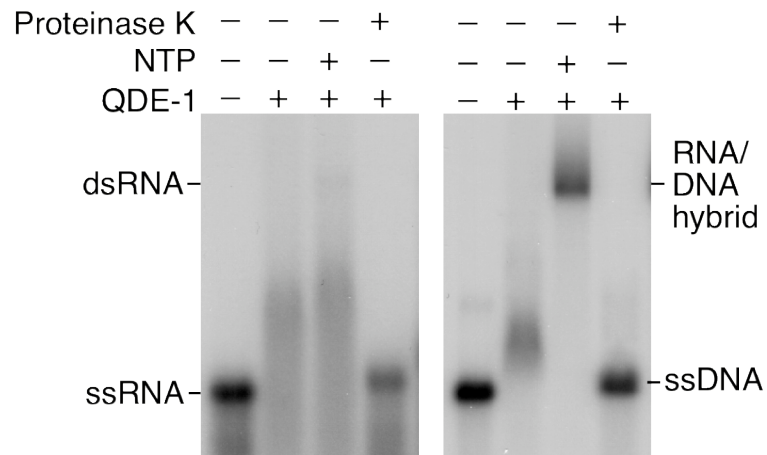

**B**

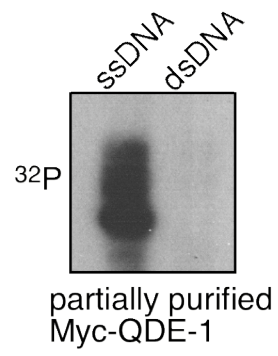

Supplement: Figure S3 — (A) RNA polymerase reactions showing the association of QDE-1 with ssRNA and ssDNA in the presence and absence of NTP. 32P-labeled ssRNA (left panel) and ssDNA (right panel) templates were used. (B) RNA polymerase reactions using Myc-QDE-1 purified from Neurospora showing that QDE-1 cannot use dsDNA as a template. (0.47 MB PDF) [file pbio.1000496.s003.pdf]
